# Time course of blast-induced injury in the rat auditory cortex

**DOI:** 10.1371/journal.pone.0193389

**Published:** 2018-02-28

**Authors:** Srinivasu Kallakuri, Edward Pace, Huichao Lu, Hao Luo, John Cavanaugh, Jinsheng Zhang

**Affiliations:** 1 Department of Biomedical Engineering, Wayne State University College of Engineering, Detroit, Michigan, United States of America; 2 Department of Otolaryngology-Head and Neck Surgery, Wayne State University School of Medicine, Detroit, Michigan, United States of America; 3 Department of Communication Sciences & Disorders, Wayne State University College of Liberal Arts & Sciences, Detroit, Michigan, United States of America; University of Florida, UNITED STATES

## Abstract

Blast exposure is an increasingly significant health hazard and can have a range of debilitating effects, including auditory dysfunction and traumatic brain injury. To assist in the development of effective treatments, a greater understanding of the mechanisms of blast-induced auditory damage and dysfunction, especially in the central nervous system, is critical. To elucidate this area, we subjected rats to a unilateral blast exposure at 22 psi, measured their auditory brainstem responses (ABRs), and histologically processed their brains at 1 day, 1 month, and 3-month survival time points. The left and right auditory cortices was assessed for astrocytic reactivity and axonal degenerative changes using glial fibrillary acidic protein immunoreactivity and a silver impregnation technique, respectively. Although only unilateral hearing loss was induced, astrocytosis was bilaterally elevated at 1 month post-blast exposure compared to shams, and showed a positive trend of elevation at 3 months post-blast. Axonal degeneration, on the other hand, appeared to be more robust at 1 day and 3 months post-blast. Interestingly, while ABR threshold shifts recovered by the 1 and 3-month time-points, a positive correlation was observed between rats’ astrocyte counts at 1 month post-blast and their threshold shifts at 1 day post-blast. Taken together, our findings suggest that central auditory damage may have occurred due to biomechanical forces from the blast shockwave, and that different indicators/types of damage may manifest over different timelines.

## Introduction

Blast exposure, which can be characterized by high wave pressure change and high energy impulse noise, as well as the related traumatic brain injury (TBI), has become a common threat in modern war theaters. Studies have found that up to 15.8% of combat-injured participants had TBI [1], and that blast exposure was the predominant cause for their TBI [2]. Individuals with blast-related TBI can experience a range of disability, including motor deficits, cognitive decline, and notably, auditory dysfunction. Blast-related TBI and auditory dysfunction is especially important considering that up to 67% of blast-exposed individuals with mild TBI have significant hearing threshold shifts and 59% develop tinnitus [3]. Presently, there are no universally-effective treatments for individuals suffering from these problems.

A major step in improving treatment for blast-related TBI and auditory dysfunction is to gain a better understanding of their underlying mechanisms. Using animal models, blast exposure has been shown to induce neurodegeneration in the auditory cortex (AC) [4], and upregulate glial fibrillary acidic protein (GFAP), *c-fos* and axonal injury markers within the central auditory structures [5]. Previous work in our lab suggested that blast induced both damage and compensatory changes in the inferior colliculus and the medial geniculate body of rats [6], as well as increased activity across the auditory pathway and limbic system [7]. We also showed that rats with blast-induced tinnitus experience increased spontaneous activity of the dorsal cochlear nucleus and inferior colliculus between 1 day and 1 month following blast exposure [8, 9], and increased activity in the AC at 3 months post-exposure [10]. Indeed, blast exposure can induce a wide range of effects with various time courses in the brain, including potentially lasting effects such as elevated GFAP expression [11], and axonal injury [12]. Further investigation of lasting neurotrauma is vital since it may directly subserve chronic, blast-induced dysfunction.

Despite the knowledge gleaned in the aforementioned work, many individuals continue to suffer from blast-related auditory dysfunction, such as tinnitus, hearing problems, and more. Thus, there is a need to further delineate the underlying mechanisms, which will help facilitate the development of more effective, customized treatment strategies for patients. Specifically, there is a need to investigate the time course of blast-induced auditory damage and related TBI. We intended to answer a question of whether blast-induced damage in the auditory cortex is predominantly caused by the direct mechanical compression and shearing of brain tissues from the blast shockwave, rather than by degeneration resulting from blast-induced damage via the auditory pathways. To address these issues, we set out to blast-expose rats with one ear occluded and assessed both the left and right ACs for reactive astrocytosis and axonal degeneration. We also measured hearing threshold changes by collecting auditory brainstem responses (ABRs) and assessed whether these threshold changes correlated with astrocytosis.

## Materials and methods

### Animal subjects

Twenty-seven adult male Sprague Dawley rats (381±15.8 grams) purchased from Envigo (formerly Harlan Laboratories, Indianapolis, IN) were used for these experiments. These same rats, combined with additional rats, were used as part of our previously published neurophysiological studies [8–10]. The rats were either blast-exposed and retained for survival periods of 1 day (n = 5), 1 month (n = 7) or 3 months (n = 8), or underwent a sham procedure (n = 6). All procedures were approved by the Institutional Animal Care and Use Committee (IACUC) and followed the Federal Animal Welfare Act.

### Blast overpressure induction

Experimental rats were exposed to a single 22 psi blast (152 kPa, 197.5 dB SPL) generated by a shock-tube (ORA Inc. Fredericksburg, VA). In preparation for blast exposure, a rat was anesthetized by a mixture of isoflurane (3%) and 0.6 L/min of oxygen for a total duration of 6 minutes. While anesthetized, a rat was placed in supportive netting attached to a pole with a locking device. The rat was positioned in a rostral cephalic orientation towards the imminent shockwave. The rat’s right ear was plugged with silicone putty (Mack’s®, McKeon Products, Warren, MI) and mineral oil to enable unilateral, auditory blast exposure. Peak static overpressure was produced with compressed helium and calibrated Mylar sheets (GE Richards Graphics Supplies Inc., Landsville, PA). Sham rats underwent the same procedures minus the blast exposure.

### Auditory brainstem responses (ABRs)

Click and tone-burst ABRs were collected in both ears prior to and after blast exposure. Rats were anesthetized with air (1 liter/min) and isoflurane (1–2.5% v/v). Subdermal recording needle electrodes were inserted, with the active electrode placed on the vertex, the reference electrode placed below the pinna, and the ground electrode placed in the contralateral temporal muscle. Click and tone-burst stimuli were delivered through an electrostatic speaker inserted in the external auditory canal. The stimuli were produced by an RX6 multifunction processor using SigGenRP software (TDT system 3; Tucker-Davis Technologies, Alachua, FL).

### Immunohistochemistry and silver impregnation

At the end of its survival period, each rat was euthanized by a lethal dose of isoflurane (5% v/v) and perfused transcardially with 4% paraformaldehyde in 0.1M PB (pH 7.4). The brain was removed, post-fixed, and subsequently cryoprotected by immersion in 30% sucrose in 0.1M PB (pH 7.4). After that, 50 μm thick frozen (−22°C) serial coronal sections encompassing the AC were cut and collected in 1x PBS filled multi-well plates. Sections from the AC were collected between -4.08mm and -6.84mm from the bregma.

#### GFAP immunostaining

To assess reactive astrocytosis in the AC, 5 representative sections per animal were subjected to antigen retrieval by incubation in a citrate buffer (pH6.0) at 90°C for 1 hour. This was followed by immersion in 0.3% hydrogen peroxide to quench endogenous peroxidase activity. The sections were then incubated overnight in a mouse anti GFAP antibody (NE1015, EMD chemicals, Gibbstown, NJ) diluted in 2% normal goat serum (Vector Laboratories, Burlingame, CA), and in 1% bovine serum albumin (BSA). The sections were then incubated in biotinylated anti-mouse IgG (Vector Laboratories, Burlingame, CA) followed by exposure to Vectastain Elite ABC reagent and chromogen development by diaminobenzidine. In control incubations, normal goat serum was substituted for primary antibody. All sections were observed under a light microscope (Leica DMLB, Leica Microsystems Ltd, Heerburg, Switzerland) to visualize astrocytes. The astrocytes were quantified by collecting 10 representative x200 digital images for each section encompassing bilateral regions of the AC. The total number of identifiable astrocytes in each digital image were counted using the cell counter function in ImageJ (http://rsb.info.nih.gov/ij/) by a blinded investigator.

#### Silver staining for degenerating axons

For qualitative analysis of blast-induced axonal injury in sections encompassing the AC, a separate set of 5 representative sections from each brain underwent a silver impregnation procedure. The sections were immersed for 3 min in pretreatment solution (equal volumes of 9% sodium hydroxide and 15% hydroxylamine) followed by a wash in 0.5% acetic acid (3x3 min) or until the sections turned opaque. The sections were then incubated in an impregnation solution (5 mg/ml ferric nitrate and 100 mg/ml silver nitrate) for 30 min. Following this, they were washed in 1% citric acid (4x2 min) followed by a wash in 0.5% acetic acid for 5 min. They were then placed in a developer solution until they turned pale gray. After sufficient development, they were removed and washed thoroughly in 0.5% acetic acid (3x10 min), rinsed in distilled water, mounted on a slide and cover-slipped, and examined under a light microscope (Leica DMLB, Leica Microsystems Ltd, Heerburg, Switzerland) for the presence of degenerating axons.

### Data analysis

ABR thresholds were defined as the lowest sound level in which part of the biological waveform was visible. The average number of GFAP reactive astrocytes in the left and right auditory cortices was determined for each animal. Astrocytosis was then compared between blast-exposed and sham animals. Qualitative analysis of axonal degeneration via silver staining was accomplished by visually examining slides for damage, such as swelling, vacuoles, and retraction balls. Representative blast-exposed and sham brain slices were compared to measure blast induced damage. Statistical comparisons between the blast-exposed and control groups were conducted using independent-samples t-tests for GFAP data, and with repeated measures ANOVA for ABR data. Greenhouse Geisser corrections were used when Mauchly’s test of sphericity was violated, and Bonferroni adjustments were made for multiple comparisons. Correlation between ABR thresholds and quantitative astrocytosis was determined using Pearson’s r. All p values were set to < 0.05 for statistical significance.

## Results

### ABR threshold changes following blast impact

**Fig 1:**
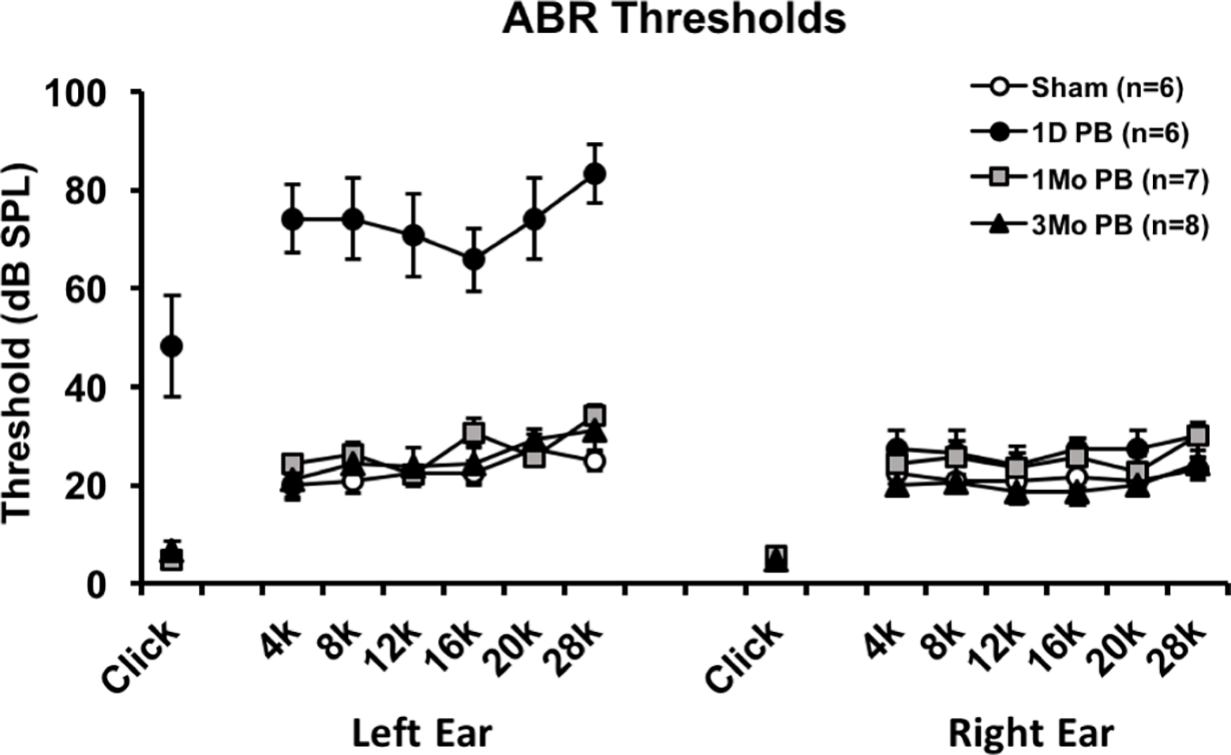
Auditory brainstem response thresholds in blast- and sham-exposed rats show temporary, blast-induced threshold elevations. For the 1 day post-blast group (1 D PB), significant threshold shifts were observed across clicks and all tone-burst frequencies in the blast-exposed ear (left ear). For the 1- and 3-month post-blast groups (1 Mo PB and 3 Mo PB), however, thresholds were similar to those of sham-exposed rats. There were no significant threshold shifts in the protected ear (right ear) following blast exposure at any time point. Error bars represent standard error of the mean.

For the 1-day post-blast group, ABR thresholds were elevated in the exposed ears (left ears) compared to sham groups’ ears (*F*_(3,23)_ = 36.097, *p <* 0.05; Fig 1). For animals in the 1- and 3-month post-blast groups, however, thresholds were similar to the sham group. Since the 1-month and 3-month animals had threshold elevations at 1 day following their respective blasts (data not shown), their thresholds had thus recovered by the 1- and 3-month time points. Thresholds in the protected ears (right ears) were not significantly different compared to shams.

### GFAP reactive astrocytosis in the AC

**Fig 2:**
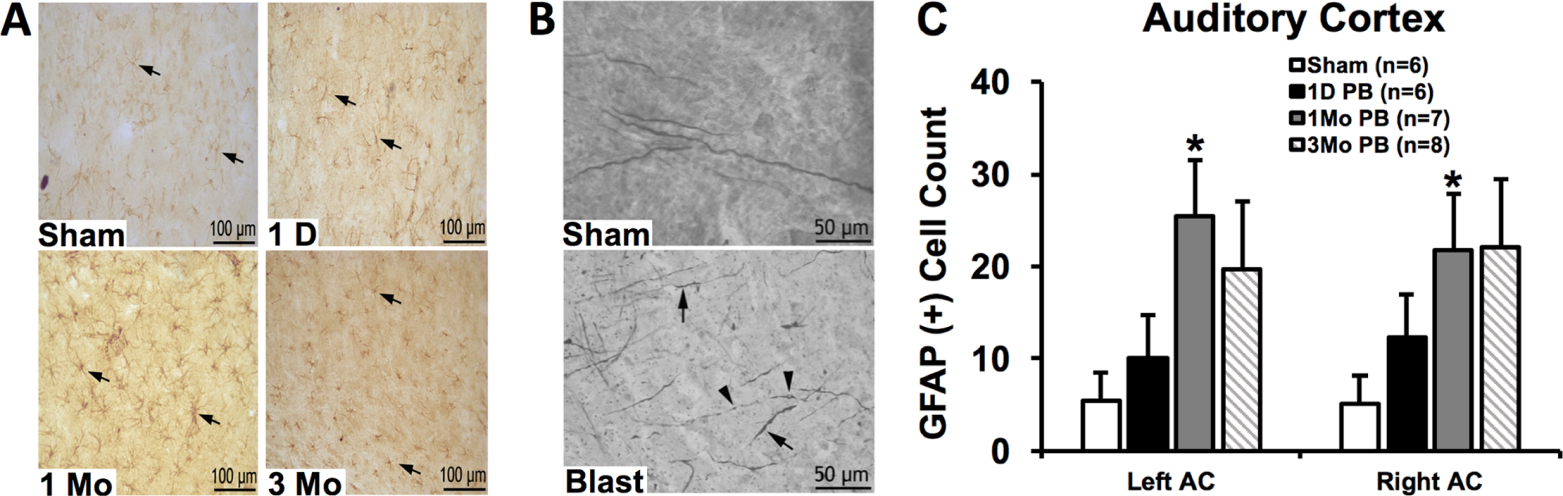
GFAP reactive astrocytosis and silver staining show central auditory damage following blast. (A) Sections encompassing the AC display GFAP reactive astrocytosis for the sham group and the post-blast day 1 (1D PB), post-blast 1 month (1 Mo PB), and post-blast 3 months (3 Mo PB) groups. (B) Silver staining revealed axons with uniform caliber in the sham group, and swollen axons (arrows) and axons with vacuoles and retraction balls (arrow heads) in the blast-exposed group. (C) Quantitative analysis of GFAP reactive astrocytosis was also conducted by comparing blast-exposed groups to the sham group. While there was little change in astrocyte counts for the 1 day post-blast group, there was a significant, bilateral increase for the 1 month post-blast group, and an insignificant increase for the 3-month post-blast group. Error bars represent standard error of the mean. * Indicates significance (p < 0.05) compared to sham group.

We compared reactive astrocytosis in the left and right ACs between sham animals and blast-exposed animals at 1 day, 1 month, and 3 months post-blast. Fig 2A shows representative sections of astrocytes from sham and blasted animals at the different time points. For quantitative analysis (Fig 2C), while the number of astrocytes were only modestly elevated in 1-day post-blast animals compared to shams, we found a significantly greater number of astrocytes in 1 month post-blast animals compared to shams in both the left AC (*t* [11] =  2.77; p  =  0.018) and right AC (*t* [10] =  2.35; p  =  0.04). Astrocytosis also showed a trend of elevation in 3 months post-blast animals, though it was not statistically significant for both the left AC (*t* [12] =  1.61; p  =  0.134) or the right AC (*t* [12] =  1.80; p  =  0.098).

### Axonal injury in the AC

Silver stained representative sections encompassing the AC of blast-exposed and sham animals were investigated for the presence of axonal damage (Fig 2B). Sham AC sections revealed long tracts of axons with uniform caliber. For blast-exposed brain sections encompassing the AC, degenerative changes in the form of axonal swelling, retraction balls, and vacuoles were observed. This degeneration was predominantly found in sections at 1 day and 3 months after blast.

### Correlation between ABR threshold and astrocytosis

**Fig 3:**
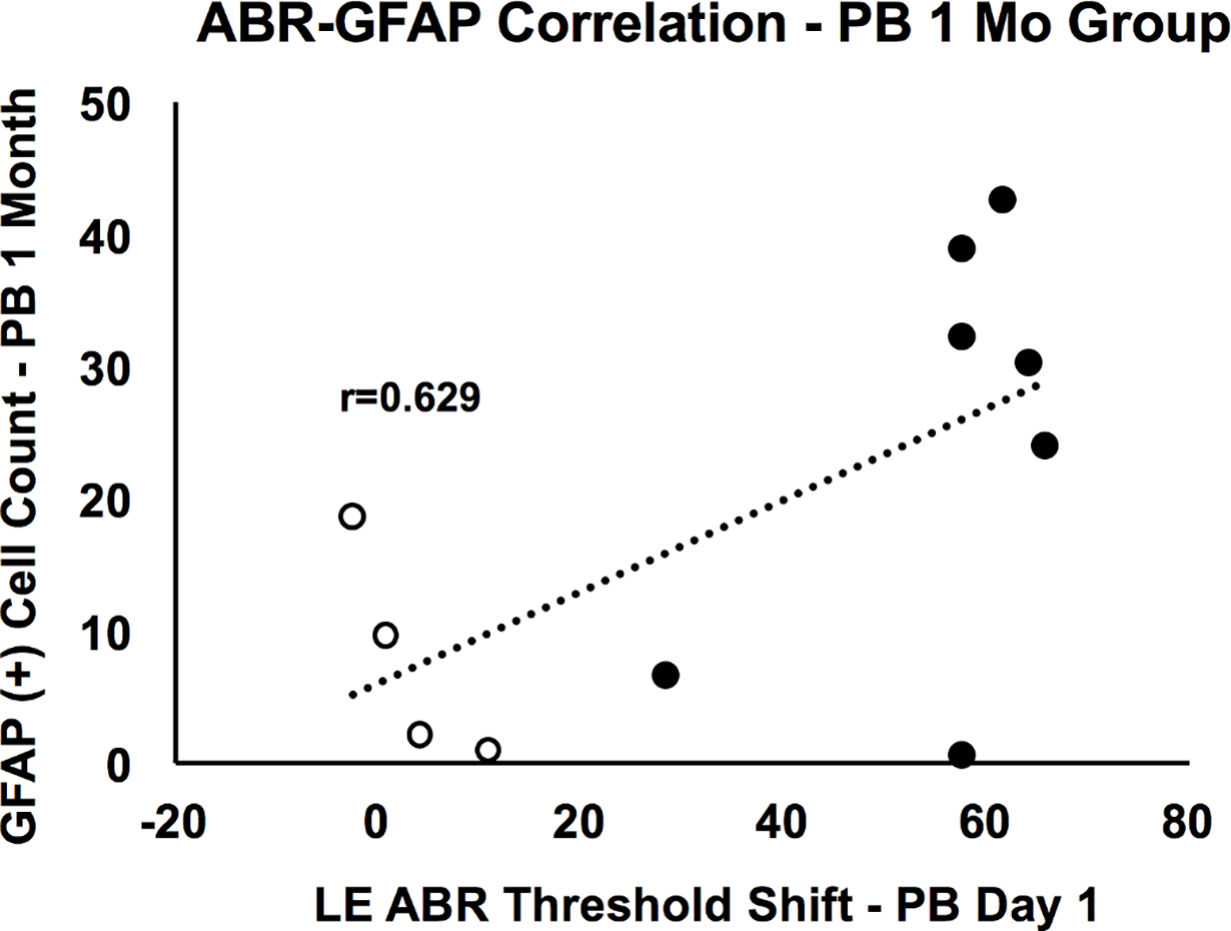
Correlation between post-blast day 1 ABR thresholds and averaged bilateral astrocyte counts of the 1-month group. A significant positive correlation was observed between ABR thresholds and astrocyte counts (p = 0.04), suggesting a putative relationship between initial hearing loss and later-manifesting central auditory damage.

ABR threshold shifts (left ears) were also tested for correlations with astrocyte counts (Fig 3). While shifts were not correlated with astrocytosis at matching time points (i.e. 1 month hearing thresholds vs. 1 month astrocyte counts), we found a significant and positive correlation between the post-blast day 1 threshold shifts of the 1-month group and the 1 month group’s averaged bilateral astrocyte counts (r = 0.63; p = 0.04).

## Discussion

To shed light on putative injury changes in the central auditory system following blast exposure, we evaluated ABR thresholds, astrocytosis and axonal integrity in the ACs. Over a period of three months following unilateral blast exposure, we observed temporary threshold shifts and axonal degeneration, followed by astrocytosis and recurring axonal degeneration. In addition, we saw a potential correlation between certain threshold shifts and astrocytosis. These findings confirm that blast exposure impacts the peripheral and central auditory system, and may suggest that brain compression and shearing forces from the blast itself play a predominant role in related auditory impairment.

ABR revealed significant hearing threshold elevations following blast in the exposed ears (left ears) of the 1-day group, but not in the unexposed ears (right ears). This demonstrates that a unilateral induction of hearing loss occurred. However, thresholds recovered to and remained at baseline levels by the 1-month and 3-month recordings. The threshold elevation and recovery timeline may be influenced by the parameters of our blast exposure (single blast at 22 psi/152 kPa) and the tone-burst frequencies (4 to 28 kHz) we used to generate ABR thresholds. Other studies have also shown that a single blast in the range of 94–150 kilopascals induced temporary ABR threshold shifts below 30 kHz [6, 9, 13]. Hearing thresholds may stay elevated for 2 weeks following blast exposure higher than 94 kPa [13], but they can remain permanently elevated for frequencies higher than 30 kHz [13]. Furthermore, while blast-induced tympanic membrane perforation has been observed in rodents and is reversible, perforations alone appear insufficient to account for the degree of blast-induced hearing threshold shifts [13]. However, it has been demonstrated that temporary threshold shift is associated with swelling of cochlear nerve terminals at their hair cell synapses [14, 15], buckling of pillar bodies [16] as well as synaptopathy [17, 18].

When assessing for GFAP reactive astrocytosis, we found significant numbers of astrocytes in both the left and right ACs of the 1 month post-blast rats, as well as a trend of elevation in the 3 month post-blast rats, compared to shams. However, there was relatively little elevation in the 1 day post-blast group. Thus, astrocytosis in the ACs following blast exposure may occur in a time-dependent manner. Others have also implicated a non-uniform time course, in which GFAP was upregulated at 0–6 hours after blast, showed no upregulation at 48 hours post-blast, and then showed upregulation again at 72 hours post-blast [19]. Long-term GFAP upregulation has also been shown at 1 to 3 months post-blast, depending on the brain structure analyzed [20]. This may be due to continuing neuronal loss, which may in part be contributed by pathologic tau protein aggregation in the ACs after blast exposure [21].

Mixed findings in GFAP expression after blast exposure have been reported by others [5, 19, 21–23]. One study, for example, found that GFAP-positive cell densities were not significantly elevated in the AC at 21 days following three blast exposures at 14 psi [5]. It is possible that a single blast of a higher intensity may be more damaging in some ways than multiple blasts of lower intensity. For example, one study found that intracranial pressure trended higher in rats exposed to a single 110 kPa blast versus rats exposed to three 72 kPa blasts [24]. Since GFAP appears to play an important role in responding to increased intracranial pressure [25], this could explain why we saw significant GFAP accumulation whereas the study employing multiple lower PSI blasts did not. Interestingly, it should be noted that some other studies employing more intense blasts (35–52 PSI) than the current study also did not observe significant GFAP expression in the cortex [11, 22], though they did not focus on the auditory cortex. One explanation for these results is that certain forms of blast-induced damage, such as those that induce GFAP expression, might peak at a certain intensity of blast and actually reduce at higher intensities. This can be seen in one study, where GFAP expression in the cortex was only significantly higher in response to 17 PSI at 0, 6, and 72 hours post-blast, and not at other time points and not in response to 14 PSI *or* 22 PSI blasts 19]. This may result from different biomechanical responses of the skull to different shockwave parameters and blast exposure setups; a specific range of loading may induce higher vulnerability to certain types of blast-induced injury, especially given the nonlinear, viscoelastic nature of the skull and brain’s response to the shock wave. It’s also possible that only certain cortical regions manifest blast-induced astrocytosis, and only at certain post-injury time points. Indeed, some studies have found increased GFAP expression in the prefrontal cortex from anywhere between 7 days to 3 months following blast [20,23].

Qualitative analysis of the axons encompassing the AC showed degradation primarily at 1 day and 3 months post-blast. Axonal disconnection can occur within hours following blast exposure [26], which correlates with the axonal degeneration that we observed at 1 day post-blast. An interesting result within our study, however, was that axonal degeneration was negligible by 1 month post-blast, but then turned prominent again at 3 months post-blast. Like reactive astrocytosis, there may be a time-dependent variable in blast-induced axonal degeneration. Noise exposure studies, while not the same as blast, have shown that while axonal degeneration initially occurs within 1–16 weeks, it may later become self-sustaining, with fresh degeneration occurring up at 8 months post-injury [27]. In a previous DTI study conducted in our lab, we saw diffusion changes that actually suggested increased axonal integrity at 2 and 4 weeks post-blast [6], which has also been found in the auditory midbrain of humans with hearing loss [28]. It seems possible, therefore, that initial damage can be sustained, followed by recovery or compensatory processes, which may then be followed by a slower-occurring axonal degradation. The latter may have been seen in one diffusion tensor imaging study, where age-related decline in white matter integrity appeared exacerbated in blast-exposed individuals [29].

Although axonal degeneration did not parallel GFAP accumulation in our study, others have also shown that axonal degeneration and GFAP accumulation are not always correlated [5, 22]. Understanding the underlying mechanisms is complicated by the fact that astrocytes can produce both growth-promoting and inhibitory effects [30]. On one hand, it is known that after a 72 hour period following axonal degeneration, the glial response (including astrocytes and macrophages) may help clear axonal debris and facilitate regeneration [31]. Potentially, this played a role in restoring axonal integrity, as we observed by the 1 month post-blast time point. On the other hand, once major central nervous system damage has occurred, reactive astrocytes can promote dense gliotic scarring that can inhibit axonal regeneration. It is thus possible that in response to continuous, long-term neuronal loss and central nervous system damage following blast and blast-related TBI, sufficient gliotic scarring occurred such that sometime after 1 month post-blast, axonal integrity could no longer be maintained. Further work is needed to delineate the mechanisms and time course of this sophisticated process, and might help to inform and improve therapeutic intervention.

An important finding of the current study is that while we limited blast-induced hearing loss to one ear, we found increased astrocytosis in both the left and right ACs. This raises the possibility that the blast shockwave, including shearing and compression forces on the brain, plays a major role in central auditory damage and related dysfunction. This builds upon our recent findings, where we used manganese-enhanced MRI to show that unilateral blast exposure can raise activity in both the left and right auditory pathways, and even in the limbic system [7]. Other studies have shown that blast exposure can damage the corpus callosum [5, 32] and white matter areas in brainstem and cerebellum [22, 33], demonstrating a diffuse effect that may not be specific to the auditory system alone. Furthermore, studies of non-blast TBI have also reported evidence of auditory dysfunction [34], astrocytosis in the cortex [35] and corticospinal axonal degeneration [36]. This role for blast-related TBI in the central auditory system is an important distinction from other inducers of central auditory damage, such as noise trauma. Understanding the similarities and differences of blast-related auditory TBI may be pivotal to determining the underlying mechanisms of and best treatment for related dysfunction, such as hearing loss and tinnitus.

While traumatic noise alone can induce astrocytosis in the cochlear nucleus [37], as well as axonal degeneration in the cochlear nucleus, superior olivary complex, and inferior colliculus [27, 37, 38], it’s less clear if this also occurs at the level of the auditory cortex. One study found sparser concentration of degenerating terminal axons in the superior olivary complex and inferior colliculus than in the cochlear nuclei following noise exposure, suggesting that regions higher along the auditory pathway are more resistant to such damage [27]. Furthermore, if the intense noise of the blast was the primary driver of the observed astrocytosis in the current study, one would expect a greater impact on the right auditory cortex compared to the left cortex. While this renders calls into question the correlation between the 1 month group’s astrocyte counts and their day 1 ABR threshold shifts, others have shown a significant association between tympanic membrane perforation and concussive brain injury [39]. It is therefore feasible that other peripheral mechanisms of hearing loss, such as swelling of nerve terminals, may correlate with later-manifesting central nervous system pathology. One wonders whether the use of standardized tools like ABR may help forecast prognosis, later-onset injury patterns, and guide the timeline of treatment intervention.

Ultimately, further work is still needed to firmly distinguish which aspects of blast-induced injury are due to the intense noise of the blast, and which are TBI due to biomechanical forces from the blast shockwave. Additional investigation would be beneficial, such as plugging both ears during blast and further examination of the specific effects on ipsilateral versus contralateral auditory pathways following noise and blast. Potentially, damage that occurs through different mechanisms may also have different characteristics, and their identification may yield better treatment of auditory dysfunction through targeted therapeutic strategies.

## Supporting information

S1 Fig Data behind Fig 1. Auditory brainstem response thresholds in blast- and sham-exposed rats show temporary, blast-induced threshold elevations.
(XLSX)

S2 Fig Data behind Fig 2. GFAP reactive astrocytosis and silver staining show central auditory damage following blast.
(XLSX)

S3 Fig Data behind Fig 3. Correlation between post-blast day 1 ABR thresholds and averaged bilateral astrocyte counts of the 1-month group.
(XLSX)
